# Hidradenitis Suppurativa as a Potential Subtype of Autoinflammatory Keratinization Disease

**DOI:** 10.3389/fimmu.2020.00847

**Published:** 2020-05-20

**Authors:** Toshifumi Nomura

**Affiliations:** Department of Dermatology, Faculty of Medicine and Graduate School of Medicine, Hokkaido University, Sapporo, Japan

**Keywords:** hidradenitis suppurativa, autoinflammatory keratinization disease, γ-secretase, Notch signaling, interleukin 1β, interleukin 17, inflammasome, biologics

## Abstract

Hidradenitis suppurativa (HS) is a chronic inflammatory skin condition, clinically characterized by boiled cysts, comedones, abscesses, hypertrophic scars, and/or sinus tracts typically in the apocrine-gland-rich areas such as the axillae, groin, and/or buttocks. Although its precise pathogenic mechanisms remain unknown, I herein emphasize the importance of the following three recent discoveries in the pathogenesis of HS: First, heterozygous loss-of-function mutations in the genes encoding γ-secretase, including *NCSTN, PSENEN*, and *PSEN1*, have been identified in some patients with HS. Such genetic alterations result in hyperkeratosis, dysregulated hair follicle differentiation, and cyst formation via aberrant Notch signaling. Furthermore, *Psen1–*/*Psen2*–, *Psen1*–, *Ncstn*+/–, and *Notch1–*/*Notch2–* mice share common phenotypes of human HS, suggesting a role of aberrant keratinization in the development of HS. Second, upregulation of interleukin 1β, interleukin-36, caspase-1, and NLRP3 and dysregulation of the Th17:Treg cell axis have been demonstrated in HS samples, suggesting that autoinflammation is a key event in the pathophysiology of the disease. Notably, HS may be complicated with other autoinflammatory diseases such as inflammatory bowel diseases and pyoderma gangrenosum, again highlighting the importance of autoinflammation in HS. Last, biologics such as adalimumab, infliximab, anakinra, ustekinumab, and secukinumab are reportedly effective for moderate-to-severe HS. These findings collectively suggest that HS is closely linked with aberrant keratinization and autoinflammation, raising the question whether it represents an autoinflammatory keratinization disease, a recently proposed disease entity. In this mini review, I introduce the concept of autoinflammatory keratinization disease and attempt to address this clinically important question.

## Introduction

Autoinflammatory keratinization diseases (AIKDs) belong to a recently proposed disease concept defined by the following four points (1). First, the primary and main inflammation sites are the epidermis and the upper dermis (1). Second, inflammation in the epidermis and upper dermis leads to hyperkeratosis, which is the main and characteristic phenotype of AIKDs (1). Third, AIKDs have primary genetic causative factors associated with the hyperactivation of innate immunity (autoinflammation), mainly in the epidermis and upper dermis (1). Last, the concept of AIKDs encompasses diseases with mixed pathomechanisms of autoinflammation and autoimmunity (1). In the original report of AIKDs, this novel disease category encompasses several genetic skin diseases caused by mutations in *CARD14, IL36RN*, or *NLRP1* (1). It was recently proposed that hidradenitis suppurativa (HS) and porokeratosis should also be categorized as AIKDs (2–4). This mini review aims to provide a concise overview highlighting the aberrantly keratinizing and autoinflammatory nature of HS and to discuss whether it represents an AIKD.

## Clinicopathological Features and Epidemiology of HS

HS, also known as acne inversa, is a chronic inflammatory skin disease of the hair follicle that usually presents after puberty, with a recurrent and progressive disease course (5–8). Clinical features of HS vary in severity and may include inflamed cysts, comedones, papules, pustules, nodules, abscesses, hypertrophic scars, fistulae, and tunneling sinus tracts, most commonly distributed in the apocrine-gland-rich and intertriginous areas such as the axillae, groin, perineum, buttocks, medial thighs, inframammary folds, and postauricular regions (5–8). Patients with HS may experience pain, pruritus, chronic malodorous purulent discharge, scar contracture, and/or sexual dysfunction and distress (5–9). Thus, HS often causes both physical and psychosocial burdens and severely impairs patients' quality of life (9–11).

There is a preponderance of females among HS patients, with an estimated female-to-male ratio of 2–3:1 (12–14). The previously published prevalence estimates of HS vary greatly from 0.05% to 4.1% depending on the types of studies (8, 13, 14); the lower estimates are derived from registry studies and the higher ones from self-reported studies (8). The exact prevalence of HS remains unknown, because, due to the hidden nature of the disease, it is an under-reported condition. Surveys show that the mean delay in the diagnosis of HS is 7.2 years (15), which may result from a lack of awareness of HS or the absence of internationally recognized diagnostic criteria (16). The diagnosis is usually made for a clinical history of recurrent, painful, inflammatory lesions in characteristic apocrine-gland-bearing areas (16).

HS was originally considered a bacterial skin infection in apocrine sweat glands because of the clinical features such as purulent discharge and the common involvement of the apocrine-gland-bearing areas. However, microbiologic screening usually reveals negative cultures or the detection of mixed normal flora and skin commensals as the main bacteria cultured from suppurative discharge (7). Notably, in a histological study of axillary skin excised from 12 patients with HS, the majority of cases (10 out of 12) showed cystic epithelium-lined structures or sinus tracts lined by squamous epithelium, both of which are derived from hair follicles (17). In contrast, only 4 out of 12 cases displayed inflammation in the apocrine glands (17). In another histological study of 60 HS biopsy samples, major findings included follicular occlusion (17/60), folliculitis (17/60), sinus tracts (9/60), epithelial cyst (6/60), and abscess (5/60) (18). Taken together, HS is now regarded as a non-suppurative disease of the hair follicle—rather than a simple bacterial infection—that is characterized by follicular occlusion or cyst formation.

## Mutations in *NCSTN, PSENEN*, and *PSEN1* are Responsible for HS

Approximately 34–42% of patients with HS report a family history of the condition, showing an autosomal dominant inheritance pattern (19–21). In 2010, heterozygous loss-of-function mutations in *NCSTN, PSENEN*, and *PSEN1* were identified in six Chinese patients with HS (22). These genes encode components of γ-secretase, an intramembrane protease that cleaves various substrates, including Notch receptors. Subsequent studies in multiple populations such as British, French, African-American, Japanese, and Chinese have robustly confirmed the pathogenic role of these genes in HS patients with a positive family history of the disease (3, 19, 23–29). Interestingly, disease-causing variants have also been identified in four non-familial, sporadic cases. However, the frequency of identifying pathogenic variants in these genes is rare—~5% of overall HS cases (7)—even in familial HS cases. Furthermore, no significant genotype–phenotype correlation has been reported so far (30). Although γ-secretase is composed of presenilin, presenilin enhancer-2, nicastrin, and anterior pharynx defective encoded by *PSEN1*/*PSEN2, PSENEN, NCSTN*, and *APH1A*/*APH1B*, respectively (31), no disease-causing mutations in *PSEN2, APH1A*, or *APH1B* have been identified in HS patients (16). Notably, in the clinical trial of γ-secretase inhibitor nirogacestat in 17 adults, six exhibited follicular and cystic lesions in intertriginous regions (32). Furthermore, mice models such as *Psen1–*/*Psen2*–, *Psen1*–, and *Ncstn*+/– mice show follicular keratinization, cyst formation, epidermal hyperplasia, follicular atrophy, and/or absent sebaceous glands, which recapitulate the histological features of HS in humans (33, 34). Mice with pharmacologic inhibition of γ-secretase also develop HS-like lesions observed in *Ncstn*+/– mice (34). These human and mice data indicate that haploinsufficiency of the γ-secretase components, though rare, plays a key role in the development of HS.

## Deficient Notch Signaling Plays a Key Role in HS

The Notch pathway regulates cell proliferation, cell differentiation, and cell death in many organs, including the skin (22, 33, 34). Importantly, the discovery of disease-causing mutations in *NCSTN, PSENEN*, and *PSEN1* in HS patients strongly suggests that haploinsufficiency of the γ-secretase components cause HS via reduced Notch signaling (22). To date, more lines of evidence have pointed to the causal role of deficient Notch signaling in HS. First, the phenotypes of *Notch1–*/*Notch2–* mice mirror those of *Psen1–*/*Psen2*– mice, showing epidermal cysts and hyperkeratosis of the follicular epithelium with the occlusion and dilatation of the follicle by keratin plug formation (33). Second, mutations in *POFUT1* and *POGLUT1* cause HS and/or Dowling–Degos disease, an autosomal dominant skin disease clinically characterized by flexural hyperpigmentation (35, 36). Given that *POFUT1* and *POGLUT1* encode GDP-fucose protein O-fucosyltransferase 1 and protein O-glucosyltransferase 1, respectively, both of which are involved in the Notch pathway (35, 36), there should be a causal link between HS and reduced Notch signaling. Furthermore, mutations in *PSENEN* have also been reported in patients with HS and/or Dowling–Degos disease (37–39), which indicates that haploinsufficiency of presenilin enhancer-2 due to *PSENEN* mutations and reduced Notch signaling due to *POFUT1* and *POGLUT1* mutations both result in similar clinical consequences. These studies have placed deficient Notch signaling at the center of HS pathogenesis.

## More Genes Associated With HS

Several HS or HS-like cases have been described in association with pachyonychia congenita or steatocystoma multiplex caused by mutations in *KRT17* or *KRT6A* (40, 41). Mutations in *FGFR2* also underlie nevus comedonicus and HS-like skin lesions (42). Furthermore, four cases of keratitis–ichthyosis–deafness syndrome, an autosomal-dominant skin disease caused by mutations in *GJB2* and occurring in association with the follicular occlusion triad (HS, acne conglobata, and dissecting folliculitis of the scalp) have been reported (43–45). These findings further highlight the importance of aberrant cyst formation and hair follicle occlusion in the pathophysiology of HS.

HS can present as a component of systemic autoinflammatory syndromes like pyoderma gangrenosum, acne, pyogenic arthritis, and HS (PAPASH) and pyoderma gangrenosum, acne, and HS (PASH), which are caused by mutations in *PSTPIP1* (46, 47). HS may also be presented by patients with familial Mediterranean fever carrying *MEFV* mutations (48–50). Notably, the frequency of *MEFV* mutations in the group of patients with HS was higher than that in healthy controls (49), suggesting that *MEFV* mutations may contribute to the pathogenesis of HS. Genetic variants in other autoinflammatory genes (e.g., *NOD2, LPIN2, NLRP3, NLRP12, PSMB8, MVK, IL1RN*) have also been identified in patients with HS or its syndromic forms (51). Thus, autoinflammation is the other key event in the pathogenesis of HS.

These findings collectively suggest that gene mutations leading to aberrant differentiation of the hair follicle epithelium and autoinflammation are associated with HS.

## Immunological Features of HS

The precise mechanism leading to inflammation in patients with HS has not been fully elucidated. However, it is thought that dermal seeding of keratin, sebum, bacterial components, and cellular debris can result in a dense infiltration of immune cells consisting of T cells (mainly CD4+, but also CD8+), B cells, macrophages, and neutrophils in HS lesional skin (52). One of the most remarkable immunological features observed in HS lesioned skin is the marked upregulation of interleukin 1β (IL-1β) (53), mainly secreted by macrophages, the most numerous inflammatory cells found in HS infiltrates (54). Highly activated IL-1β pathways induce the strong production of chemokines (e.g., CXCL1, CXCL6), which contributes to the massive infiltration of immune cells, including neutrophils, thereby leading to clinical features of HS such as purulent discharge (53). Furthermore, IL-1β enhances the secretion of matrix metalloproteinases such as MMP3 and MMP10, which appear to be involved in tissue destruction, another major feature of HS (53). Notably, the increased expression of caspase-1, NLRP3, IL-6, IL-18, and IL-36 has also been reported (53). These findings emphasize the role of autoinflammation in the pathophysiology of HS.

Other major immunological characteristics of HS include the upregulation of IL-17 and tumor necrosis factor-α (51–53, 55, 56). The enrichment of Th17 cells and the dysregulation of the Th17:Treg cell axis, likely driven largely by increased IL-1β and IL-6 as a result of inflammasome activation (55), are remarkable features seen in HS lesioned skin (55, 56). Notably, the impaired Th17/Treg balance has also been shown in various autoinflammatory diseases, including inflammatory bowel diseases, Behçet's disease, and spondyloarthritis (56). These findings further indicate that HS has an autoinflammatory nature.

## Biologics for HS

Given the immunological features of HS as discussed above, one could envision that immunomodulatory treatments targeting cytokines such as IL-1, IL-17, and tumor necrosis factor-α are beneficial (57). Indeed, the efficacy of biologics such as adalimumab, infliximab, etanercept, anakinra, ustekinumab, and secukinumab have been reported for moderate-to-severe HS (57–60). Particularly, adalimumab is the only biologic approved for this indication worldwide at the time of submission of this manuscript. In two phase 3 randomized controlled trials, adalimumab, 40 mg weekly, achieved significantly higher clinical response rates at week 12 than the placebo in patients with moderate-to-severe HS (58). This positive effect has lasted through week 168 (59). Notably, Moran et al. (55) showed that inhibition of tumor necrosis factor-α led to the reduction of IL-17 and the normalization of a dysregulated Th17:Treg cell axis in the skin, which may explain its therapeutic effects in HS. The significant response to biologics further supports the notion that HS should be considered a disease of aberrant immunity and not simply a bacterial infection.

## Conclusion and Future Perspectives

**Figure 1:**
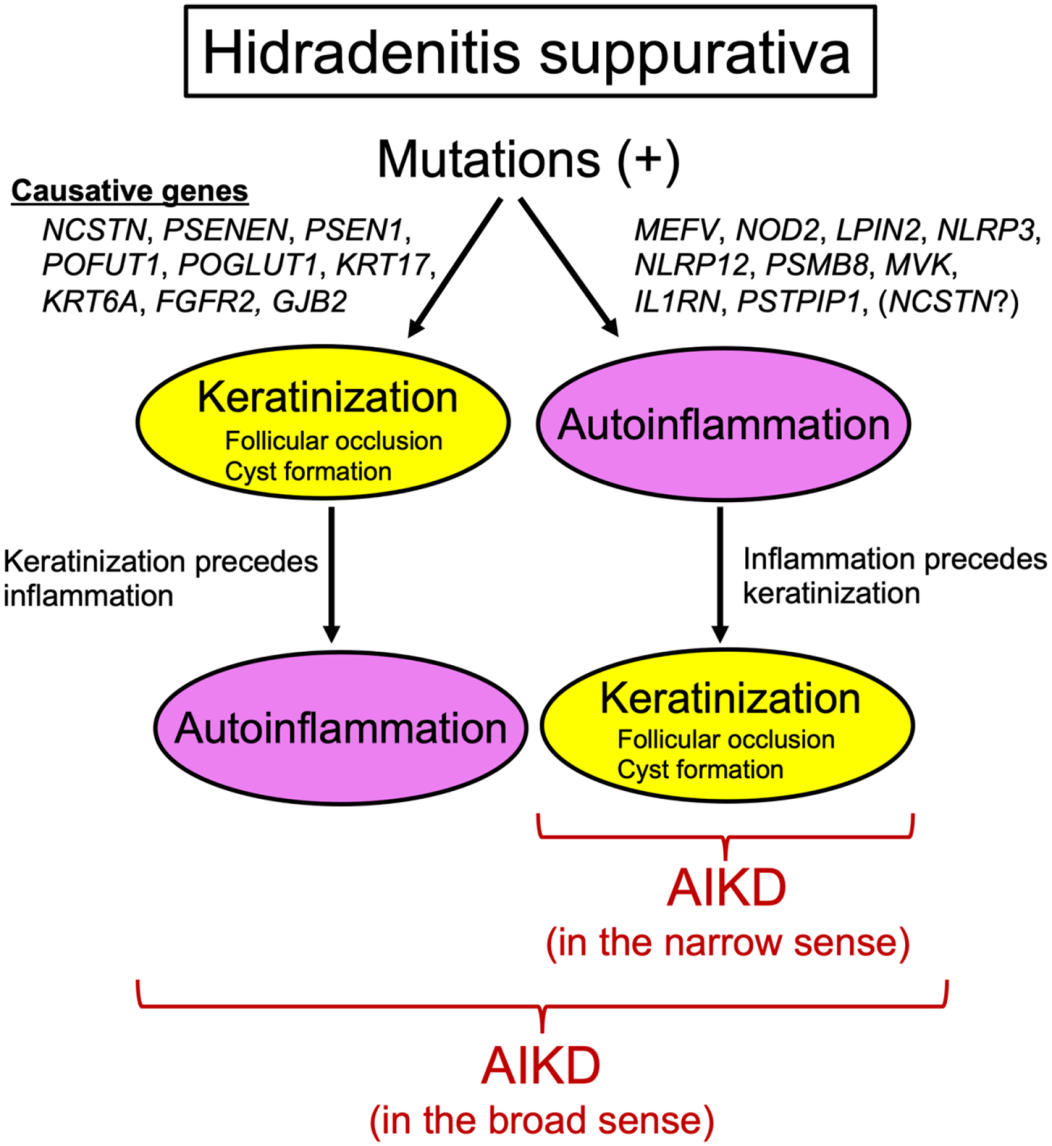
Hidradenitis suppurativa and autoinflammatory keratinization disease. Genes responsible for hidradenitis suppurativa can be divided into two groups. One includes *NCSTN, PSENEN, PSEN1, POFUT1, POGLUT1, KRT17, KRT6A, FGFR2*, and *GJB2*, whose mutations result in autoinflammation preceded by keratinization. This hidradenitis suppurativa subtype can be regarded as an autoinflammatory keratinization disease in the broad sense. The other group includes *MEFV, NOD2, LPIN2, NLRP3, NLRP12, PSMB8, MVK, IL1RN, PSTPIP1*, and possibly *NCSTN*, whose mutations lead to keratinization preceded by autoinflammation. This hidradenitis suppurativa subtype can be regarded as an autoinflammatory keratinization disease in the narrow sense.

The recent findings discussed above collectively suggest that HS is closely linked with aberrant keratinization and autoinflammation and thereby that, in the broad sense, HS should be regarded as an AIKD (Figure 1). However, caution should be taken in reaching a definitive conclusion as to whether HS should be included as an AIKD in the narrow sense (Figure 1) because it is uncertain whether the inflammatory response precedes the hyperkeratotic events such as hyperkeratosis of the terminal hair follicle epithelium or cyst formation in HS. It is generally believed that follicular occlusion is the primary event in HS. However, given that variants in autoinflammatory genes (e.g., *MEFV, NOD2, LPIN2, NLRP3, NLRP12, PSMB8, MVK, IL1RN, PSTPIP1*), which should lead to hyperkeratosis preceded by inflammation, have been linked to HS phenotypes (49) and that a loss-of-function mutation in *NCSTN* was identified in one PASH patient (61), one could predict that at least some HS patients completely fulfill all of the AIKD criteria proposed by Akiyama et al. (1) (Figure 1). Future research on the molecular basis of HS, especially capturing the full picture of the immune milieu and identifying more responsible genes, should address the question at hand and may benefit patients with this intractable disease.

## Author Contributions

TN conceived the work, performed the literature review, and wrote the manuscript.

## Conflict of Interest

The author declares that the research was conducted in the absence of any commercial or financial relationships that could be construed as a potential conflict of interest.

## Abbreviations

HS: hidradenitis suppurativa
AIKDs: autoinflammatory keratinization diseases
PAPASH, pyoderma gangrenosum, acne, pyogenic arthritis: and HS
PASH, pyoderma gangrenosum, acne: and HS
IL-1β: interleukin 1β.
